# AB0/Rhesus Blood Group Does Not Influence Clinicopathological Tumor Characteristics or Oncological Outcome in Patients Undergoing Radical Prostatectomy

**DOI:** 10.3389/fsurg.2017.00075

**Published:** 2017-12-18

**Authors:** Su Jung Oh, Philipp Mandel, Felix K. H. Chun, Pierre Tennstedt, Sven Peine, Jan Lukas Hohenhorst, Jens Hiller, Markus Graefen, Derya Tilki, Thomas Steuber

**Affiliations:** ^1^Martini Klinik Prostate Cancer Center, University Hospital Hamburg-Eppendorf, Hamburg, Germany; ^2^Department of Anatomy and Experimental Morphology, University Hospital Hamburg-Eppendorf, Hamburg, Germany; ^3^Department of Urology, University Hospital Hamburg-Eppendorf, Hamburg, Germany; ^4^Department of Transfusion Medicine, University Hospital Hamburg-Eppendorf, Hamburg, Germany; ^5^Department of Urology, Pediatric Urology and Urologic Oncology, Kliniken Essen-Mitte, Essen, Germany

**Keywords:** blood groups, cancer risk, outcome, prostate cancer, radical prostatectomy

## Abstract

**Objectives:**

AB0 blood group is an inherited characteristic that has been associated with the incidence as well as the prognosis of several malignancies. The aim of the current study was to clarify the role of the blood group in cancer epidemiology and clinical outcome of patients with prostate cancer (PCa).

**Methods:**

Data from 3,574 patients undergoing radical prostatectomy between 2009 and 2010 at a single European institution were retrospectively analyzed. The correlation of AB0 and Rhesus blood group with PCa-related characteristics and oncological outcome were evaluated using univariable and multivariable Cox proportional hazard models.

**Results:**

Median follow-up was 36.9 months. The overall distributions of AB0, as well as Rhesus blood groups among patients with PCa, did not differ from the distribution observed in the normal population. There was no significant association between AB0/Rhesus blood groups and Gleason score, prostate volume, surgical margin, pT-stage, pN-status, or preoperative prostate-specific antigen level. In multivariable Cox regression analysis, no statistically significant correlation between AB0/Rhesus group and biochemical recurrence was observed (all *p* > 0.05).

**Conclusion:**

Our data suggest no relevant association of AB0/Rhesus blood group with adverse clinicopathological tumor characteristics or oncological outcome after surgery in contrast to several other malignancies.

## Introduction

Prostate cancer (PCa) is the most common malignancy in Western countries and the second leading cause of cancer-related deaths in males (1). The most recognized risk factors for developing PCa are increasing age, ethnic origin, and family history (2). The familial predisposition suggests an inherited genetic component to the disease (3), but accounts for only a minority (5–10%) of PCa cases. As a consequence, predisposing environmental and genetic factors for most patients are still unknown (4, 5). AB0 blood type is an inherited characteristic that has been associated with the incidence as well as the prognosis of several malignancies (6–8). Several plausible mechanisms, including inflammation, immune-surveillance for malignant cells, intercellular adhesion, and membrane signaling have been proposed to explain the observed association between AB0 blood groups and cancer risk.

AB0 blood group antigens (ABH) are glycoproteins expressed on the surface of red blood cells. The phenotypic A and B antigens are terminal carbohydrates synthesized by the addition of single sugars catalyzed by a series of specific glycosyltransferases. Phenotype 0 is characterized by the absence of A and B glycosyltransferases, so that only a protein backbone, the H antigen, is present. Red blood cell antigens have various functions, including membrane structural integrity, transportation of molecules through membranes, and adhesion (9). Along with their expression on red blood cells, ABH antigens are also expressed in a variety of epithelial cells including urothelium, gastrointestinal mucosa, and lungs. Several studies demonstrated changes of ABH expression pattern in the cancer cells by tumor grade and disease progression suggesting a possible association between AB0 blood group and the risk of some epithelial malignancies (8, 10). Recent studies demonstrated that individuals with blood group A, AB, or B had an increased incidence of pancreatic cancer compared to those with blood group 0 while the highest risk among was found to be related to blood group B (8). Similarly, blood serotype A was reported to be associated with higher risk of gastric, breast and ovary cancer (11). On the other hand, blood group 0 was correlated with the highest recurrence and progression rates after transurethral bladder resection and thus associated with a worse prognosis in patients with non-muscle invasive bladder urothelial carcinoma (6).

Based on the current evidence of the predictive role of the blood group in several malignancies, we hypothesized that the type of blood group may be used as an additional parameter, which facilitates the assessment of risks and helps to estimate prognosis after surgery in patients with PCa. In current literature, there is only limited clinical evidence available regarding blood group and PCa in general, which demonstrated controversial results (12–14) Moreover, currently, only one study with a special focus on the impact of blood groups on the outcome in patients after radical prostatectomy (RP) exists (13). Using 555 patients with PCa undergoing RP, the authors showed AB0 blood group to be an independent predictor of biochemical recurrence (BCR) in the multivariable analysis but not in univariable analysis.

Therefore, the aim of the present study was to add further information to the ongoing debate by analyzing the correlation of AB0/Rh blood group and its distribution within patients with PCa, tumor pathology, recurrence, and mortality in patients with localized PCa undergoing RP by using a large single center dataset of 3,574 patients.

## Materials and Methods

### Study Population

The study was approved by the institutional review board. A total of 3,582 patients with clinically localized PCa who underwent RP at our institution between 2009 and 2010 were screened for study inclusion. Those with incomplete data sets and missing follow-up were excluded (*n* = 8), leaving 3,574 patients available for analysis.

Radical prostatectomy was performed using an open retropubic approach (*n* = 3,220) or robotic-assisted laparoscopic approach (*n* = 354), as described previously (15). Lymph node dissection was performed in D’Amico intermediate-risk and high-risk patients according to the guidelines of the European Association of Urology (EAU) for PCa (16). Surgical specimens were processed according to standard histopathological procedures and evaluated by experienced uropathologists in a high-volume center (>2,000 prostatectomy specimens per year). Tumors were staged according to the 2007 American Joint Committee on Cancer TNM staging system and histopathological grading was assigned according to the Gleason system (17).

All participants underwent serologic testing prior to RP. Laboratory confirmation of AB0 and Rh blood group were obtained from the Department of Transfusion Medicine, University Hospital Hamburg—Eppendorf. The AB0 reference distribution of the German population was used as control (18).

### Study Variables

Study variables included AB0/Rh blood group, age, preoperative prostate-specific antigen (PSA), prostate volume (transrectal ultrasound), Gleason score (specimen), pT-stage, pN-status, surgical margin, BCR, and mortality after RP. Patient follow-up consisted of PSA testing every 3–6 months during the first 2 years after RP, BCR was defined as PSA level ≥0.2 ng/ml.

The relationship between AB0 blood group and clinicopathological variables were assessed using chi-squared tests and *t*-tests. In Cox regression multivariable analysis (log-rank tests), the impact of blood group, preoperative PSA, Gleason score, pT-stage, pN-status, and surgical margin on BCR-free survival were assessed in order to evaluate the prognostic values for survival. The probability of BCR-free survival was compared in 0, A, B, and AB groups using Kaplan–Meier analysis and the log-rank test.

All tests were two-tailed and *p*-values <0.05 were considered statistically significant. Statistical analyses were performed with JMP software v9.0.2 (SAS Institute, Inc., Cary, NC, USA) and R v2.13.1 (R Project for Statistical Computing, www.R-project.org).

## Results

A total of 3,574 patients with clinically localized PCa were eligible for final analyses, the median age at surgery was 65 years (range: 38–80) and the median follow-up was 36.9 months (Table 1).

**Table 1:** Association of the AB0 blood group and Rhesus factor with clinicopathologic characteristics of 3,574 patients treated with radical prostatectomy.

	Overall (*n* = 3,574)	AB0 blood group					Rhesus factor		
		0	A	B	AB	*p*-Value	RH negative	RH positive	*p*-Value
					
*N* (%)		1,350 (37.8)	1,605 (44.9)	445 (12.5)	174 (4.9)		603 (16.9)	2,971 (83.1)	
**Age at surgery**									
Median	65	65	59	59	61	0.15	65	65	0.69
IQR	59; 69	60; 65	59; 69	59; 69	61; 69		60; 69	59; 69	
**Prostate volume (TRUS)**									
Median	40	39	40	39	41	0.45	40	40	0.79
IQR	30; 52	30; 51	30; 53	30; 52	31; 55		30; 53	30; 52	
**Rh factor**									
Negative	603 (16.9)	194 (14.4)	292 (18.2)	79 (17.8)	38 (21.8)	**0.01**			
Positive	2,971 (83.1)	1,156 (85.6)	1,313 (81.8)	366 (82.2)	136 (78.2)				
**Preoperative prostate-specific antigen (ng/ml)**									
<4	386 (10.8)	154(11.4)	169 (10.6)	42 (9.4)	21 (12.1)	0.55	67 (11.1)	319 (10.8)	0.94
4–10	2,262 (63.3)	841 (62.4)	1,030 (64.4)	292 (65.6)	99 (57.2)		376 (62.4)	1,886 (63.7)	
10–20	672 (18.8)	250 (18.6)	303 (18.9)	79 (17.8)	40 (23.1)		118 (19.6)	554 (18.7)	
>20	245 (6.9)	102 (7.6)	98 (6.1)	32 (7.2)	13 (7.5)		42 (7)	203 (6.9)	
**Gleason score (specimen)**									
3 + 3	473 (13.2)	190 (14.1)	201 (12.6)	58 (13)	24 (13.8)	0.77	88 (14.6)	385 (13)	0.60
3 + 4	2,391 (66.9)	881 (65.4)	1,094 (68.4)	304 (68.3)	112 (64.4)		395 (65.7)	1,996 (67.3)	
4 + 3	532 (14.9)	203 (15.1)	234 (14.6)	66 (14.8)	29 (16.7)		93 (15.5)	439 (14.8)	
≥4 + 4	169 (4.7)	73 (5.4)	70 (4.4)	17 (3.8)	9 (5.2)		25 (4.2)	144 (4.9)	
**pT-stage**									
pT2	2,454 (68.7)	901 (66.7)	1,135 (70.9)	305 (68.5)	113 (64.9)	**0.03**	402 (66.8)	2,052 (69.1)	0.34
pT3a	699 (19.6)	279 (20.7)	308 (19.2)	79 (17.8)	33 (19)		131 (21.8)	568 (19.1)	
≥pT3b	417 (11.7)	170 (12.6)	158 (9.9)	61 (13.7)	28 (16.1)		69 (11.5)	348 (11.7)	
**pN-status**									
pNx	1,038 (29)	392 (29.1)	464 (29)	133 (29.9)	49 (28.2)	0.79	166 (27.7)	872 (29.4)	0.48
pN0	2,303 (64.4)	865 (64.2)	1,044 (65.3)	279 (62.7)	115 (66.1)		400 (66.7)	1,903 (64.2)	
pN+	224 (6.3)	91 (6.8)	90 (5.6)	33 (7.4)	10 (5.7)		34 (5.7)	190 (6.4)	
**Surgical margin**									
R0	2,904 (81.3)	1,082 (80.1)	1,320 (82.5)	361 (81.1)	141 (81)	0.44	487 (80.9)	2,417 (81.5)	0.75
R1	665 (18.6)	268 (19.9)	280 (17.5)	84 (18.9)	33 (19)		115 (19.1)	550 (18.5)	
**Follow-up (months)**									
Median	36.9	36.9	36.9	36.9	37.6	0.90	36.9	36.9	0.23
IQR	25.0; 48.6	25.4; 48.6	24.9; 48.6	26.5; 48.6	30.8; 48.6		24.8; 48.5	20.1; 48.7	

*N, number of patients; IQR, interquartile range*.

*Bold font indicates significance on a p < 0.05 significance level*.

### Distribution of AB0/Rh Blood Group in Patients with PCa

The distribution of AB0 blood group among patients with PCa prior to RP was as follows: 0 in 1,350 (38%), A in 1,605 (45%), B in 445 (12%), and AB in 174 (5%) patients, 83% of the patients were Rh positive (Table 1). Reference distribution for the German population is given as (18): 0 in 41%; A in 43%; B in 11%; AB in 5%; Rhesus positive: 85%. Overall distributions of AB0 as well as Rh blood groups in the study cohort were equivalent to the distribution observed in the German male population.

### No Significant Differences in Clinicopathologic Characteristics among PCa Patients According to AB0/Rh Blood Groups

The majority of patients had clinically localized PCa with intermediate risk profile. The most reported Gleason score was 3 + 4 (66.9%) and 63.3% of patients had preoperative PSA levels within the range of 4–10 ng/ml. pT2 tumors were detected in 68.7% and negative margins were achieved in 81.3% of patients. The median prostate volume was 40 ml and lymph node involvement was diagnosed in 224 (6.3%) patients (Table 1). There were no significant differences in clinicopathologic characteristics among patients with different AB0 blood groups. Slightly increased percentages of advanced tumor stage (≥pT3b) were found in patients with blood group AB compared to blood groups 0, A and B (16.1, 12.6, 9.9, and 13.7%, respectively, *p* = 0.033). Patient population with AB also showed a moderately increased ratio of negative Rh factor (21.8%) in comparison to the other blood groups (16.9%, *p* = 0.01) (Table 1).

### No Significant Prognostic Value of Blood Group for BCR-Free Survival after RP

**Figure 1:**
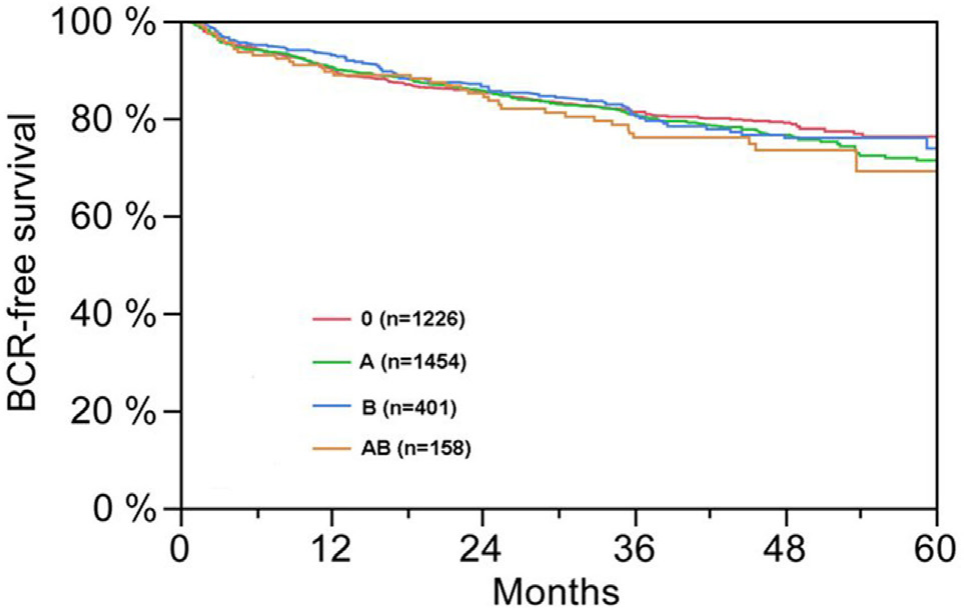
Biochemical recurrence (BCR)-free survival after radical prostatectomy stratified by AB0 blood groups (*p* = 0.572).

**Figure 2:**
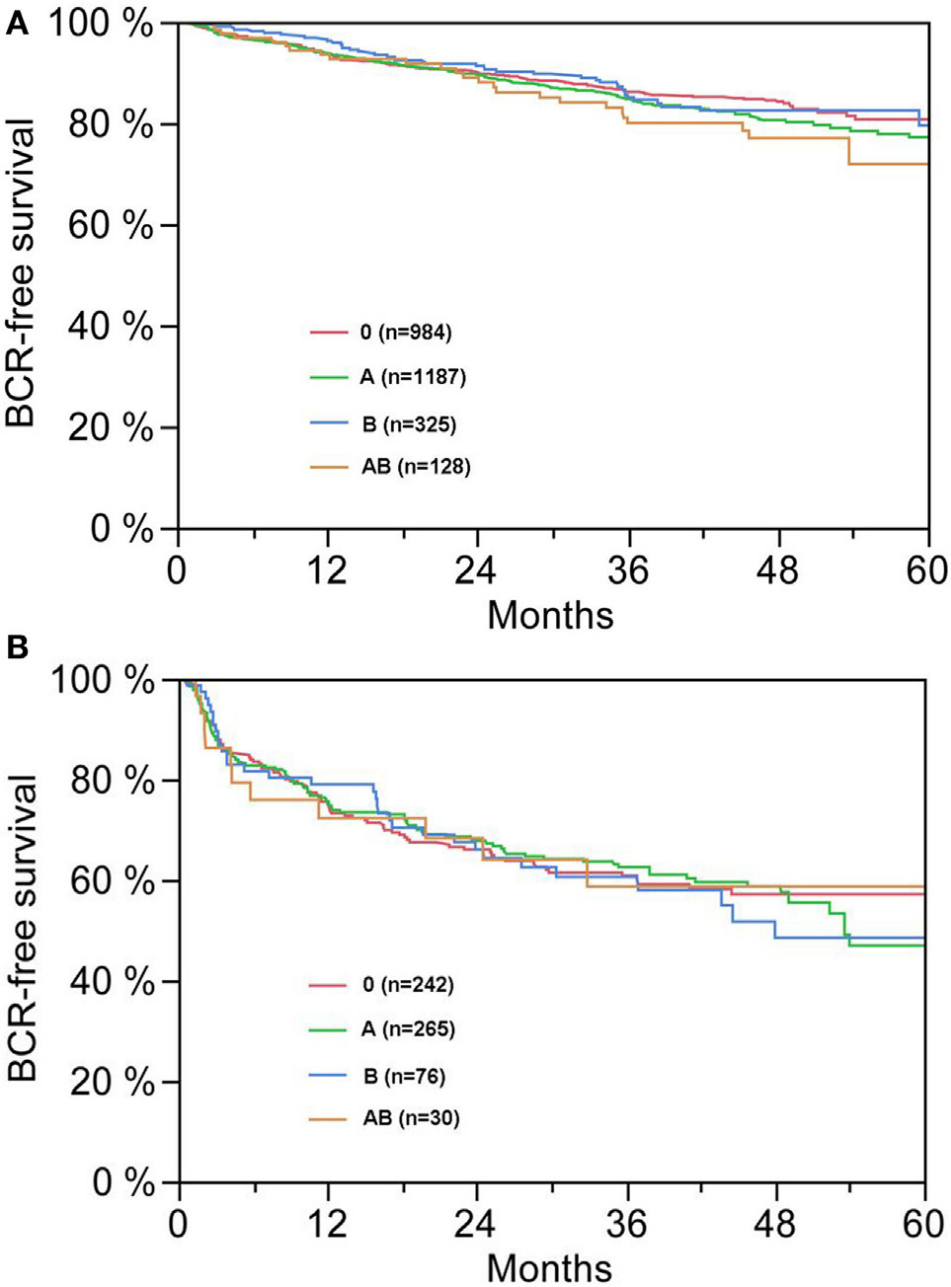
**(A,B)** Biochemical recurrence (BCR)-free survival after radical prostatectomy stratified by AB0 blood groups in patients with negative [**(A)**, *p* = 0.300] and positive [**(B)**, *p* = 0.990] surgical margin status.

According to multivariable cox regression analysis, preoperative PSA, Gleason score, pT-stage, pN status, and surgical margin significantly and independently correlated with BCR-free survival (all *p* < 0.05) (Table 2). However, we could not observe any impact of the AB0 blood group on BCR rates (Figure 1). We further assessed the prognostic value of blood group 0 compared to non-0 blood groups as well as within the alternative blood groups A/B/AB with insignificant results (Table 2). Even if subanalysis with patients with positive or negative margin status only were performed, the influence of AB0/Rh on BCR remained insignificant (Figures 2A,B). Moreover, patients’ Rhesus factor status also had no impact on BCR-free survival (*p* = 0.74).

**Table 2:** Multivariable cox regression models of AB0 and standard prognostic factors for biochemical recurrence-free survival.

Parameter	HR	CI 95%	*p*-Value
**AB0**			
A vs. 0	1.1	0.96–1.36	0.13
B vs. 0	1.0	0.76–1.28	0.96
AB vs. 0	1.2	0.81–1.65	0.38
A vs. B	1.2	0.90–1.49	0.26
A vs. AB	1.0	0.70–1.40	0.88
B vs. AB	0.8	0.57–1.27	0.41
**Preoperative prostate-specific antigen**			
4–10 vs. <4	1.4	1.01–2.08	**0.04**
10–20 vs. <4	2.3	1.59–3.36	**<0.0001**
>20 vs. <4	2.0	1.37–3.08	**0.0003**
**Gleason score (specimen)**			
3 + 4 vs. 3 + 3	1.9	1.23–3.15	**0.0034**
4 + 3 vs. 3 + 3	4.3	2.68–7.26	**<0.0001**
≥4 + 4 vs. 3 + 3	3.9	2.31–6.95	**<0.0001**
**pT-stage**			
pT3a vs. pT2	2.1	1.67–2.54	**<0.0001**
≥pT3b vs. pT2	3.0	2.29–3.85	**<0.0001**
**pN-status**			
pNx vs. pN0	0.6	0.46–0.80	**0.0002**
pN+ vs. pN0	1.7	1.34–2.18	**<0.0001**
**Surgical margin**			
R1 vs. R0	1.3	1.07–1.56	**0.0079**

*Bold font indicates significance on a p < 0.05 significance level*.

## Discussion

Possible associations between AB0 blood group and the risk of some epithelial malignancies, including pancreatic-, colorectal-, and gastric cancer have been reported frequently (19, 20). Moreover, the AB0/Rh blood groups are available for most patients prior to surgical intervention, thus suggested as an ideal adjunctive marker without any additional laboratory processing steps.

Several decades ago, immunohistochemical studies indicated that patient-derived PCa cells show different patterns of ABH expression than normal/benign prostate cells (21). Since both AB0 blood group and its antigen expression on tissue have been reported to be associated with incidence, disease progression and outcome in several malignancies (8, 22, 23), such altered expression of ABH antigen in prostate tissue suggested a possible influence of the blood group on the malignant transformation of prostate cells.

A recent study published by Markt and colleagues (14) analyzed the relationship between AB0 blood type and the risk of harboring aggressive PCa. The authors analyzed a dataset of 2,774 patients with aggressive PCa and 4,443 patients without PCa as controls. Using these data, a significant correlation between AB0 blood type or “dose” of A or B alleles and aggressive PCa risk could not be shown. While the authors also reported cancer-specific and overall mortality stratified by blood groups, the study did not include information on PCa treatment. Moreover only patients with aggressive tumors were included which might mask the potential effect of different blood groups.

These results are in line with a study run by Kvist and colleagues, which demonstrated no correlation between AB0 blood type and the survival of patients with PCa managed with watchful waiting. This study was mainly limited by the small case number (*n* = 279) (12). Interestingly, the only study focusing on patients after RP including 555 Japanese patients recently reported that blood group 0 was significantly associated with a decreased risk of BCR after RP in multivariable but not univariable analysis (13). In the subanalysis of patients with a negative surgical margin, the 5-year BCR-free rate in blood group 0 was significantly higher than that in group A (91.2 vs. 71.0%; *p* = 0.026). In this study cohort, 46.3% of patients had positive margin status despite of a clinical stage of T1c in 74.2% of the patients. The pathological stage (pT) was not mentioned.

Our retrospective cohort of 3,574 patients with clinically localized PCa is the largest cohort being analyzed so far to determine a possible impact of both AB0 and Rh blood groups on tumor characteristics and outcome after RP. In the present study, the majority of patients had pT2 tumors (68.7%) and 18.6% had positive margin status. Using these data, we could not show any significant impact of AB0/Rh blood groups on clinicopathologic characteristics (preoperative PSA, Gleason score, prostate volume, pT stage, pN status, and surgical margin) and BCR-free survival after curative RP. Moreover, when performing a subanalysis with patients with positive or negative margin status only, the influence of AB0/Rh on BCR remained insignificant. Hence, we could not reproduce the results of the Japanese study (13). In contrast to other malignancies such as pancreas or bladder cancer, AB0/Rh blood group provided no prognostic value in PCa.

There are several important limitations to our study. First and foremost are the limitations inherent to retrospective analyses and the lack of a control group. Furthermore, our study cohort predominantly contains localized PCa with low- to intermediate risk profile (Gleason score 3 + 4 in 66.9%, preoperative PSA of 4–10 ng/ml in 63.3%, pT2 in 68.7% and negative margins in 81.3% patients) allowing pre-selection of patients with favorable prognosis. In addition, the overall distribution of AB is—in contrast to the other blood groups—generally low abundant (4.9%). Some observation such as the moderately increased ratio of negative Rh factor (21.8%) in patients with AB in comparison to the other blood groups (16.9%, *p* = 0.01) could be possibly boosted due to the rarity of Rh-negative AB type, which prevents a statically significant detection of a correlation within this subgroup. Finally, information on genotype of the respective blood group was missing.

In addition to the above mentioned features, environmental, geographic, and racial concerns can never be neglected in discussing correlations of AB0 blood group with cancer. The racial and ethnic differences in blood groups as well as in PCa are well known (24, 25). In Europe, where PCa is the most common malignancy and the second leading cause of cancer related death in men (1), the most frequent blood types are A and 0, increasing to allele B from West to East. In contrast, Asian countries with the lowest incidence and low grade of PCa are characterized by a high occurrence of types B and relative low frequency of 0, as well as smaller population with negative Rh factor (26). In this respect, our results differ from the current study in Japanese patients with relative high positive margin status and BCR after curative RP (13).

Several mechanisms have been proposed to explain how blood group influences cancer incidence and progression; however, they still remain unclear. In case of bladder cancer, a chromosomal aberration was suggested, because the AB0 gene is located on chromosome 9q34, an area that is frequently alterated in bladder cancer (27). Since the AB0 gene actually encodes for glycosyltransferases, which catalyze the transfer of sugars to the H antigen (0), the occurrence of bladder cancer is potentially correlated with the blood group 0. In contrast to that, it has been reported that individuals with non-0 blood groups (A, AB, and B) have an elevated risk of developing gastric cancer and indicate a cancer specific role of the blood group.

Regarding PCa, there are several efforts underway to elucidate the role of blood group in survival after therapy. It was already reported that patients with non-0 blood type should have increased venous thromboembolism risk after RP, which could have an influence on the outcome (28). Even a recently published study demonstrated that serum antibodies to blood group A predict survival on PROSTVAC-VF (a poxvirus-based therapeutic cancer vaccine in phase III clinical trials for the treatment of advanced PCa) and should be considered as a new potentially predictive biomarker for PROSTVAC-VF (29). Although we did not observe any clear association between AB0/Rh blood group and tumor pathology and outcome in our study, such evidence still suggests a predictive role of the blood group in the PCa epidemiology and clinical outcome, especially in view of therapy response.

The present study with 3,574 patients is the largest so far analyzing the impact of AB0 or Rh blood groups on pathology and outcome after RP and adds important knowledge to the literature.

In conclusion, our data suggest no relevant association of AB0/Rhesus blood group with adverse clinicopathological tumor characteristics or oncological outcome after surgery in contrast to several other malignancies. Nevertheless, further research is indicated to assess a possible association between genotype of blood group or its antigen expression on PCa tissue and prognosis within different therapy groups including high grade or advanced PCa, which were not sufficiently reflected in our study group.

## Informed Consent

Informed consent was obtained from all individual participants included in the study.

## Ethics Statement

All procedures performed in studies involving human participants were in accordance with the ethical standards of the institutional and/or national research committee and with the 1964 Helsinki declaration and its later amendments or comparable ethical standards.

## Author Contributions

SO, DT, and TS: substantial contributions to the conception or design of the work, drafting the work, final approval of the version to be published. PM: substantial contributions to the conception or design of the work, drafting the work, final approval of the version to be published. FC: acquisition, analysis, or interpretation of data for the work, revising it critically for important intellectual content, final approval of the version to be published. PT: acquisition, analysis, or interpretation of data for the work, revising it critically for important intellectual content, final approval of the version to be published. SP: acquisition, analysis, or interpretation of data for the work, revising it critically for important intellectual content, final approval of the version to be published. JLH, JH, and MG: acquisition, analysis, or interpretation of data for the work, revising it critically for important intellectual content, final approval of the version to be published. All authors agreed to be accountable for all aspects of the work in ensuring that questions related to the accuracy or integrity of any part of the work are appropriately investigated and resolved.

## Conflict of Interest Statement

The authors declare that the research was conducted in the absence of any commercial or financial relationships that could be construed as a potential conflict of interest. The handling editor declared a shared affiliation, though no other collaboration, with several of the authors (FC, SP, JH, and DT).

## References

[1] Siegel R Ma J Zou Z Jemal A. Cancer statistics, 2014. CA Cancer J Clin (2014) 64(1):9–29. 10.3322/caac.21208 24399786

[2] Key T. Risk factors for prostate cancer. Cancer Surv (1995) 23:63–77. 7621474

[3] Langeberg WJ Isaacs WB Stanford JL. Genetic etiology of hereditary prostate cancer. Front Biosci (2007) 12:4101–10. 10.2741/2374 17485361

[4] Pinsky PF Kramer BS Reding D Buys S. Reported family history of cancer in the prostate, lung, colorectal, and ovarian cancer screening trial. Am J Epidemiol (2003) 157(9):792–9. 10.1093/aje/kwg043 12727673

[5] Lichtenstein P Holm NV Verkasalo PK Iliadou A Kaprio J Koskenvuo M Environmental and heritable factors in the causation of cancer – analyses of cohorts of twins from Sweden, Denmark, and Finland. N Engl J Med (2000) 343(2):78–85. 10.1056/NEJM200007133430201 10891514

[6] Klatte T Xylinas E Rieken M Kluth LA Roupret M Pycha A Impact of ABO blood type on outcomes in patients with primary nonmuscle invasive bladder cancer. J Urol (2014) 191(5):1238–43. 10.1016/j.juro.2013.11.106 24333243

[7] Duell EJ Bonet C Munoz X Lujan-Barroso L Weiderpass E Boutron-Ruault MC Variation at ABO histo-blood group and FUT loci and diffuse and intestinal gastric cancer risk in a European population. Int J Cancer (2015) 136(4):880–93. 10.1002/ijc.29034 24947433

[8] Wolpin BM Kraft P Gross M Helzlsouer K Bueno-de-Mesquita HB Steplowski E Pancreatic cancer risk and ABO blood group alleles: results from the pancreatic cancer cohort consortium. Cancer Res (2010) 70(3):1015–23. 10.1158/0008-5472.CAN-09-2993 20103627 PMC2943735

[9] Reid ME. The gene encoding the I blood group antigen: review of an I for an eye. Immunohematology (2004) 20(4):249–52. 15679458

[10] Nakagoe T Nanashima A Sawai T Tuji T Ohbatake M Jibiki M Expression of blood group antigens A, B and H in carcinoma tissue correlates with a poor prognosis for colorectal cancer patients. J Cancer Res Clin Oncol (2000) 126(7):375–82. 10.1007/PL00008485 10929759 PMC12165217

[11] Gates MA Wolpin BM Cramer DW Hankinson SE Tworoger SS. ABO blood group and incidence of epithelial ovarian cancer. Int J Cancer (2011) 128(2):482–6. 10.1002/ijc.25339 20309936 PMC2946962

[12] Kvist E Krogh J Hjortberg P. Prognostic variables in patients with prostate cancer: influence of blood group ABO (H), the Rhesus system, age, differentiation, tumour stage and metastases. Int Urol Nephrol (1992) 24(4):417–23. 10.1007/BF02550636 1459817

[13] Ohno Y Ohori M Nakashima J Okubo H Satake N Takizawa I Associations between ABO blood groups and biochemical recurrence after radical prostatectomy. Int J Clin Exp Med (2015) 8(2):2642–8. 25932213 PMC4402860

[14] Markt SC Shui IM Unger RH Urun Y Berg CD Black A ABO blood group alleles and prostate cancer risk: results from the breast and prostate cancer cohort consortium (BPC3). Prostate (2015) 75(15):1677–81. 10.1002/pros.23035 26268879 PMC4578997

[15] Schlomm T Tennstedt P Huxhold C Steuber T Salomon G Michl U Neurovascular structure-adjacent frozen-section examination (NeuroSAFE) increases nerve-sparing frequency and reduces positive surgical margins in open and robot-assisted laparoscopic radical prostatectomy: experience after 11,069 consecutive patients. Eur Urol (2012) 62(2):333–40. 10.1016/j.eururo.2012.04.057 22591631

[16] Mottet N Bellmunt J Briers E van den Bergh RCN Bolla M van Casteren NJ Guidelines on Prostate Cancer 2015. European Association of Urology (2015).

[17] Gleason D Mellinger G Prediction of prognosis for prostatic adenocarcinoma by combined histological grading and clinical staging. J Urol (1974) 111:58–64. 10.1016/S0022-5347(17)59889-4 4813554

[18] Pelzer U Klein F Bahra M Sinn M Dorken B Neuhaus P Blood group determinates incidence for pancreatic cancer in Germany. Front Physiol (2013) 4:118. 10.3389/fphys.2013.00118 23745115 PMC3662880

[19] Newell GR Gordon JE Monlezun AP Horwitz JS ABO blood groups and cancer. J Natl Cancer Inst (1974) 52(5):1425–30. 10.1093/jnci/52.5.1425 4831434

[20] Marcus DM The ABO and Lewis blood-group system. Immunochemistry, genetics and relation to human disease. N Engl J Med (1969) 280(18):994–1006. 10.1056/NEJM196905012801806 4888078

[21] Abel PD Marsh C Henderson D Leathem A Powell PH Williams G. Detection of blood group antigens in frozen sections of prostatic epithelium. Br J Urol (1987) 59(5):430–5. 10.1111/j.1464-410X.1987.tb04841.x 2439162

[22] Nakagoe T Fukushima K Itoyanagi N Ikuta Y Oka T Nagayasu T Expression of ABH/Lewis-related antigens as prognostic factors in patients with breast cancer. J Cancer Res Clin Oncol (2002) 128(5):257–64. 10.1007/s00432-002-0334-5 12029441 PMC12164471

[23] Orntoft TF Meldgaard P Pedersen B Wolf H. The blood group ABO gene transcript is down-regulated in human bladder tumors and growth-stimulated urothelial cell lines. Cancer Res (1996) 56(5):1031–6. 8640757

[24] Watanabe M Nakayama T Shiraishi T Stemmermann GN Yatani R. Comparative studies of prostate cancer in Japan versus the United States. A review. Urol Oncol (2000) 5(6):274–83. 10.1016/S1078-1439(00)00092-2 11008096

[25] Fontes F Severo M Castro C Lourenco S Gomes S Botelho F Model-based patterns in prostate cancer mortality worldwide. Br J Cancer (2013) 108(11):2354–66. 10.1038/bjc.2013.217 23660943 PMC3681014

[26] Garratty G Glynn SA McEntire R. ABO and Rh(D) phenotype frequencies of different racial/ethnic groups in the United States. Transfusion (2004) 44(5):703–6. 10.1111/j.1537-2995.2004.03338.x 15104651

[27] Orlow I Lacombe L Pellicer I Rabbani F Delgado R Zhang ZF Genotypic and phenotypic characterization of the histoblood group ABO(H) in primary bladder tumors. Int J Cancer (1998) 75(6):819–24. 10.1002/(SICI)1097-0215(19980316)75:6<819::AID-IJC1>3.0.CO;2-Y 9506524

[28] Clyne M Prostate cancer: non-O blood type is VTE risk factor after radical prostatectomy. Nat Rev Urol (2013) 10(12):680 10.1038/nrurol.2013.255 24189930

[29] Campbell CT Gulley JL Oyelaran O Hodge JW Schlom J Gildersleeve JC. Serum antibodies to blood group A predict survival on PROSTVAC-VF. Clin Cancer Res (2013) 19(5):1290–9. 10.1158/1078-0432.CCR-12-2478 23362327 PMC3594414

